# Altered theta oscillations in basolateral amygdala and ventral hippocampus related to social defeat

**DOI:** 10.1186/s12868-025-00972-6

**Published:** 2025-08-27

**Authors:** Xinyu Wang, Yinglong Liu, Fengkai He, Dongyong Guo, Aili Liu, Wenwen Bai, Huiyun Yang, Xinyu Xu, Xuyuan Zheng, Xiaojun Xu, TiaoTiao Liu

**Affiliations:** 1 School of Biomedical Engineering and Technology, Tianjin Medical University, Tianjin, China; 2 Department of Radiation Therapy, Tianjin Medical University General Hospital Airport Hospital, Tianjin, China; 3 Department of Anesthesiology, Tianjin Medical University Cancer Institute and Hospital, Tianjin, China; 4 School of Basic Medicine, Tianjin Medical University, Tianjin, 300070 China; 5 Bioland Laboratory, Guangzhou, Guangdong China

**Keywords:** Depression, Repeated social defeat stress, Basal lateral amygdala (BLA), Ventral hippocampus (vHPC), Theta oscillations

## Abstract

**Background:**

Depression is a prevalent mental disorder, and prolonged exposure to social defeat is a major contributing factor in the onset of depression. Repeated social defeat stress (RSDS) is a commonly used animal model for depression, significantly impacting on the pathogenesis of depression-related to social disorders. The basolateral amygdala (BLA) and the ventral hippocampus (vHPC) are critical brain regions involved in RSDS-induced social behavioral disorders, but the specific neural oscillations occurring in these regions following social defeat remain unclear.

**Methods:**

Using simultaneous multi-electrode recordings, we captured local field potentials (LFPs) from BLA and vHPC while the stressed mice underwent a social interaction test. Power spectral analysis and Amplitude transform entropy were respectively applied to assess social defeat–induced alterations in neural oscillatory activity and directional inter-regional communication.

**Results:**

Our study demonstrated that repeated social defeat induces social avoidance and depression-like behaviors. Notably, the power spectral analysis within the BLA and vHPC revealed statistically differences in the theta band (4–12 Hz) between control and RSDS groups, particularly during the With CD1 phase in the 0–3 s stage, when mice entered the social interaction zone, compared to the − 3 –0 s stage prior to enter the zone. Moreover, machine learning analysis successfully classified control and RSDS groups based on neural oscillatory activity in the BLA and vHPC. Finally, ketamine treatment was found to reduce social avoidance and depressive-like behaviors, as well as enhance theta oscillation in the BLA and vHPC.

**Conclusion:**

These results suggest that social defeat alters theta oscillations in the BLA and vHPC, highlighting potential therapeutic avenues for addressing depression-related social dysfunction.

**Supplementary Information:**

The online version contains supplementary material available at 10.1186/s12868-025-00972-6.

## Introduction

Depression, a prevalent mental disorder, typically presents with emotional symptoms such as persistent sadness, loss of interest, and diminished sense of happiness [1, 2]. Long-term repeated social defeat, often leading to social disorders, constitutes a significant factor in depression onset. Repeated social defeat stress (RSDS) stands as a widely utilized animal model of depression [3–5], characterized by social behavior disorders prominently featuring social avoidance [6, 7].

Numerous studies have shown that the basolateral amygdala (BLA) and the ventral hippocampus (vHPC) are key brain regions in social behavior disorders induced by RSDS [8, 9]. The BLA, crucial for emotion input and processing, is implicated in mood abnormalities stemming from maladaptation to social stressors [10, 11]. vHPC is responsible for emotional and motivational behavior, and plays an important role in the formation of episodic and declarative memory [12–15]. RSDS can cause a sustained increase in the activity of BLA neurons [16]. Moreover, structural plasticity occurs within the BLA and vHPC, characterized by dendritic enlargement in the BLA, positively correlated with social interaction ratios, and contrasting changes in the vHPC [17]. Additionally, neural projections from BLA to vHPC modulate social behavior [18, 19]. Activation of BLA-vHPC promotes social avoidance and reduces social interaction, while inhibition of this circuit enhances social interaction [20, 21]. Nevertheless, neural oscillations in the BLA and vHPC in response to social defeat remain elusive.

To address this issue, we employed an animal model of RSDS-induced depression-related social behavioral disorder to conduct recordings of local field potentials (LFPs) in the BLA and vHPC during a social interaction test. Subsequently, we quantitatively analyzed the neural oscillatory activity of the BLA and vHPC. The characteristics of the oscillatory activity were involved in support vector machine to classify the RSDS and control groups. Finally, Ketamine was used to ameliorate social avoidance and neural oscillations. These findings may provide a theoretical foundation for mitigating symptoms of depression-related social behavior disorder through regulation of neural oscillatory activity in the BLA and vHPC.

## Materials and methods

### Animal

All experimental procedures were performed in accordance with the Guide for the Care and Use of Laboratory Animals and approved by the Animal Care and Use Committee of Tianjin Medical University (License number: TMUaMEC2021060). Male C57BL/6J mice at 8–16 weeks of age and male CD1 retired breeder mice at 16 weeks of age were purchased from SPF Biotechnology Ltd (Beijing, China, No. SCXK 2019-0010). All CD1 mice were housed individually. All mice were maintained on a 12-h light/dark cycle at a temperature of 24 °C and a humidity of 50–55% with free access to food and water. All behavioral experiments on animals and the preparation of RSDS models were conducted during the light phase.

### Repeated social defeat stress

A modified paradigm of resident-intruder social interactions was used as described previously [5, 22]. CD1 mice were used as resident aggressors for social defeat and were individually housed prior to the experiment. Aggressive CD1 mice, exhibiting a delay of less than 60 s to the onset of initial aggressive behavior towards the intruder mice, were screened to be used in the RSDS. Experimental mice were introduced to the CD1 aggressor side of the cage and exposed to the CD1 mice for 5 min, at the end of which the experimental mice were placed on the other side of the cage. This procedure was repeated daily during the 10-day RSDS and the CD1 mice were separated from the experimental mice with clear porous Plexiglas during this period to ensure that the experimental mice had visual and olfactory contact with the CD1 mice.

### Social interaction test

Mice were placed in a novel arena (50 cm × 50 cm) containing an “interaction zone”. The interaction zone of the test arena consisted of a 14 cm × 24 cm rectangular area protruding 8 cm around the wire mesh enclosure. Mice were first free to explore for 600 s in the No target situation and then for another 600 s in the presence of CD1 mice. The arena was cleaned with 75% alcohol at the end of each stage. Mouse movements and time spent in each area were analyzed using Ethovision XT 8.5 software (Noldus Information Technology, Wageningen, Netherlands). We calculated the social interaction ratio as (time spent in the interaction zone in the presence of CD1)/(time spent in the interaction zone in the absence of CD1) [23, 24]. Only data from mice with a social interaction ratio less than 1 were included for the analysis of oscillatory activity. Neural oscillatory analysis of LFP signals encompassed a period of 3 s before and after the mice entered the social zone.

### Tail suspension test

The tail suspension test was employed to evaluate depression-like behavior in mice. Mice were affixed with tape approximately 1.5 cm from the tail’s tip and secured onto a suspension frame to induce a suspended state. Subsequently, a video camera captured the behavior of the mice in suspension for a duration of 6 min. Following the video recording, the remaining time, totaling 5 min, was measured.

### Electrode implantation surgery

The mice were anesthetized via intraperitoneal injection of pentobarbital sodium at a dose of 50 mg/kg prior to the operation and subsequently secured on a stereotactic apparatus for electrode implantation. A customized 16-channel microelectrode array [5, 25] was implanted into the BLA (−1.60 mm AP, −2.75 mm ML, −4.60 mm DV from the dura) and vHPC (−3.30 mm AP, −3.25 mm ML, −4.25 mm DV from the dura). The array was arranged in a 4 × 4 configuration: 0.033 mm diameter Nichrome wire with formvar insulation (California Fine Wire, California, USA), 0.25 mm inter-electrode spacing, impedance < 1 MΩ, and gold plating using IMP-2 A (Baker Electronics, Florida, USA). Refer to the ground wire connected to the ground screw. Finally, the electrodes were fixed to the skull with dental adhesive. Histological examination of the electrodes at the implantation site was conducted post-experiment to confirm the recording sites.

### Neurophysiological data acquisition

Neurophysiological signals from mice during the social interaction test were recorded using the Cerebus acquisition system (Blackrock Microsystems, Utah, USA). Local field potential (LFP) signals were band-pass filtered at 0.5–250 Hz and then sampled at 2 K Hz.

### Neural oscillation analysis

To determine the power spectral characteristics of the depression-related social behavioral disorder, we calculated the power spectrum density (PSD) of BLA and vHPC during “No CD1” and “With CD1” phases. First, the time-frequency analysis was carried out through the Short Time Fourier Transform (STFT), the Hamming window with a width of 1 s and a frequency of 0.5 Hz for the 3-second intervals immediately preceding and following the moment of entry into the social zone([−3 3]s). Then, the average PSD for the BLA and vHPC in five frequency rages (0.5–4 Hz, 4–12 Hz, 13–30 Hz, 30–60 Hz, 60–90 Hz) was calculated, respectively. Finally, transfer entropy is an information-theoretic measure that quantifies directed information flow between two time series [26, 27]. Amplitude transfer entropy (ATE) extends this approach to evaluate the strength and direction of connectivity between the amplitudes of neuronal oscillations, capturing how the amplitude of BLA LFPs influences that of vHPC LFPs.

### Support vector machine classification

Support Vector Machine (SVM) is a supervised machine learning model. This algorithm has found widespread application in classification tasks [5, 28, 29]. In our study, we employed an SVM model to classify the control and the RSDS groups based on the PSD features in five different frequency ranges (0.5–4 Hz, 4–12 Hz, 13–30 Hz, 30–60 Hz, 60–90 Hz) from the BLA, vHPC, and the fused features from both brain regions. The dataset was randomly divided into a training set (80%) and a test set (20%), and an SVM-based decoder was trained to distinguish between the control and the RSDS groups. To verify the accuracy of the decoding process, we repeated the training of the decoder 1000 times, with behavioral data randomly offset relative to neural activity [29]. We used accuracy, precision, F1 score, and area under the curve (AUC) to evaluate the model.

### Systemic drug delivery for antidepressant

All drugs were dissolved in 0.9% saline and administered intraperitoneally. The dose of ketamine (Hengrui Pharma) was 10 mg kg^−1^. Behavioral tests and in vivo recording were conducted on the mice at 1 h and 24 h after drug delivery [30].

### Histology

Following the completion of the electrophysiological experiment, mice were administered 1.5 times the standard anesthetic dose of pentobarbital sodium via intraperitoneal injection to achieve deep anesthesia. Subsequently, cardiac perfusion was conducted using PBS buffer and a 4% paraformaldehyde solution. Then, the brain slices of mice were obtained by freezing section and fixed on the slide. Finally, the tip of the electrode was observed and photographed.

### Statistical analysis

All data are expressed as the mean ± standard error of the mean (SEM). The statistical analyses were performed using Prism software (GraphPad 9). An unpaired t-test was used to analyze the results in the inter-group. One-way ANOVA followed by Bonferroni test for multiple comparisons was applied to compare the PSD among the different frequencies in the intra-group. Two-way ANOVA followed by Bonferroni test for multiple comparisons was applied to compare the PSD among the different stages in the intra-group. Two-way ANOVA followed by post hoc Bonferroni’s test for multiple comparisons were used to compare the accuracy, precision, F1 score and AUC of SVM. *P* < 0.05 was considered statistically significant. (*, **, and *** indicate *P <* 0.05, *P <* 0.01, and *P <* 0.001, respectively).

## Results

### Repetitive social defeat stress induces social avoidance and depression-like behaviors

**Fig. 1:**
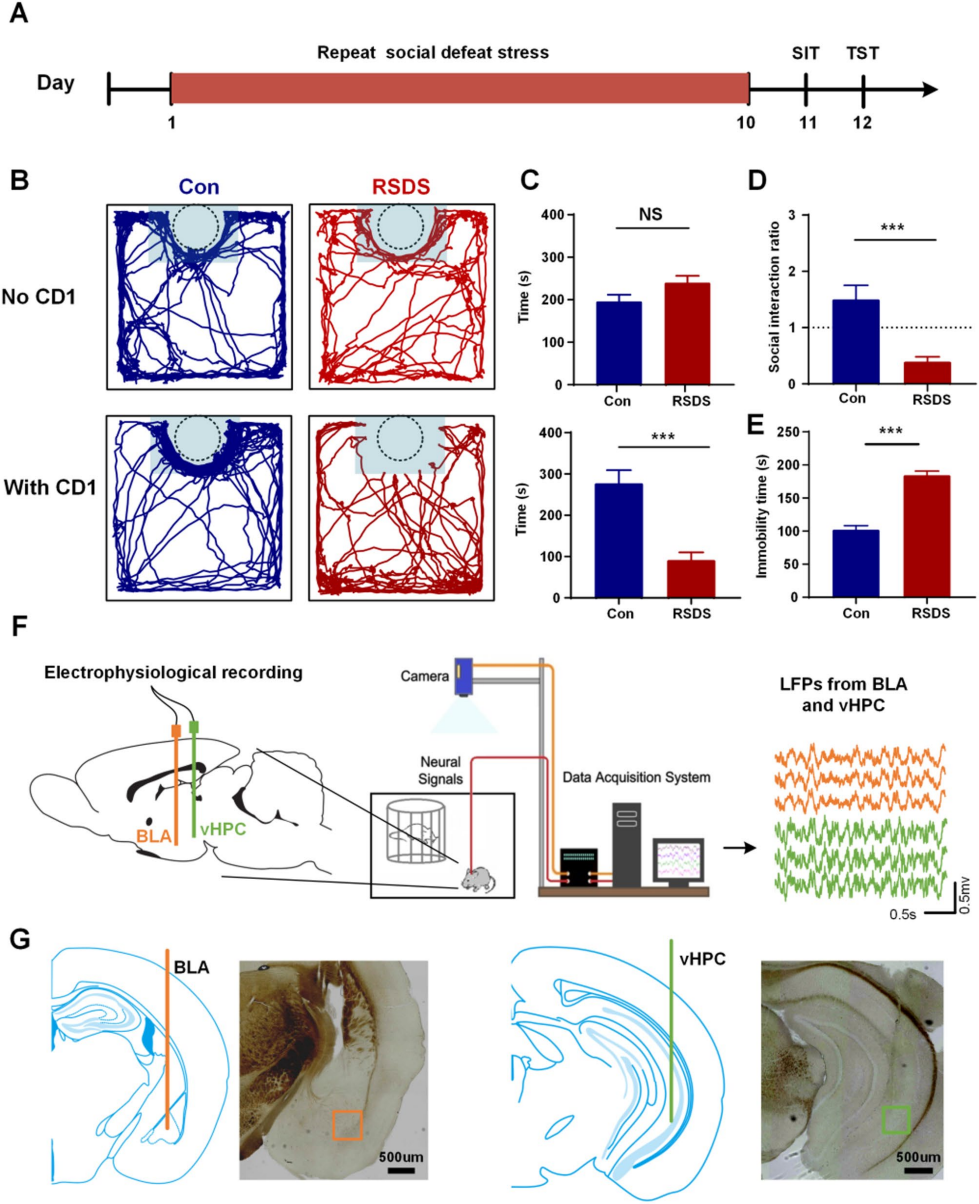
Repetitive social defeat stress induces social avoidance and depression-like behaviors. **A** Experimental timeline showing all the steps of the experimental procedure. **B** Representative activity tracking of control and social defeat mice during social interaction tests in NO CD1 and With CD1 phases. **C** Spent time in the interaction zone in No CD1 phase (top) and With CD1 phase (down). **D** Social interaction ratio in the interaction zone. **E** Immobility time in TST. **F** Schematic diagram of electrophysiological recording sites. Multichannel microelectrode arrays were implanted in the BLA and vHPC. **G** Histological verification of the recording sites in BLA (left) and vHPC (right). All data are expressed as mean ± SEM. NS, no significant; ***P < 0.001 (Con, *n* = 8; RSDS, *n* = 8)

To test the effect of the RSDS model on social behavior, SIT and TST were performed after mice were exposed to RSDS for ten days (Fig. 1A). In the SIT, social defeat mice spent significantly less time in the interaction zone when CD1 mice were present compared to controls (Con: 277.3 ± 31.83, RSDS: 91.12 ± 18.98, *P <* 0.001; Fig. 1B and C). In addition, when CD1 mice were not present, there was no significant difference in the time spent in the interaction zone by the social defeat mice compared to the controls (Con: 195.9 ± 15.81, RSDS: 239.8 ± 16.46, *p* = 0.0844;Fig. 1B and C). This suggests that exposure to repeated social defeat stress induces social avoidance behavior in mice. The social interaction ratio of the social defeat mice was significantly lower than that of the controls (Con: 1.501 ± 0.2494, RSDS: 0.3936 ± 0.08707, *P <* 0.001; Fig. 1D). In the TST, the immobility time of the social defeat mice was significantly higher than that of the control group (Con: 102.2 ± 6.050, RSDS: 184.4 ± 6.339, *P <* 0.001; Fig. 1E). These results support the validity of the RSDS model preparation.

### Social defeat alters the theta oscillations in BLA and vHPC

**Fig. 2:**
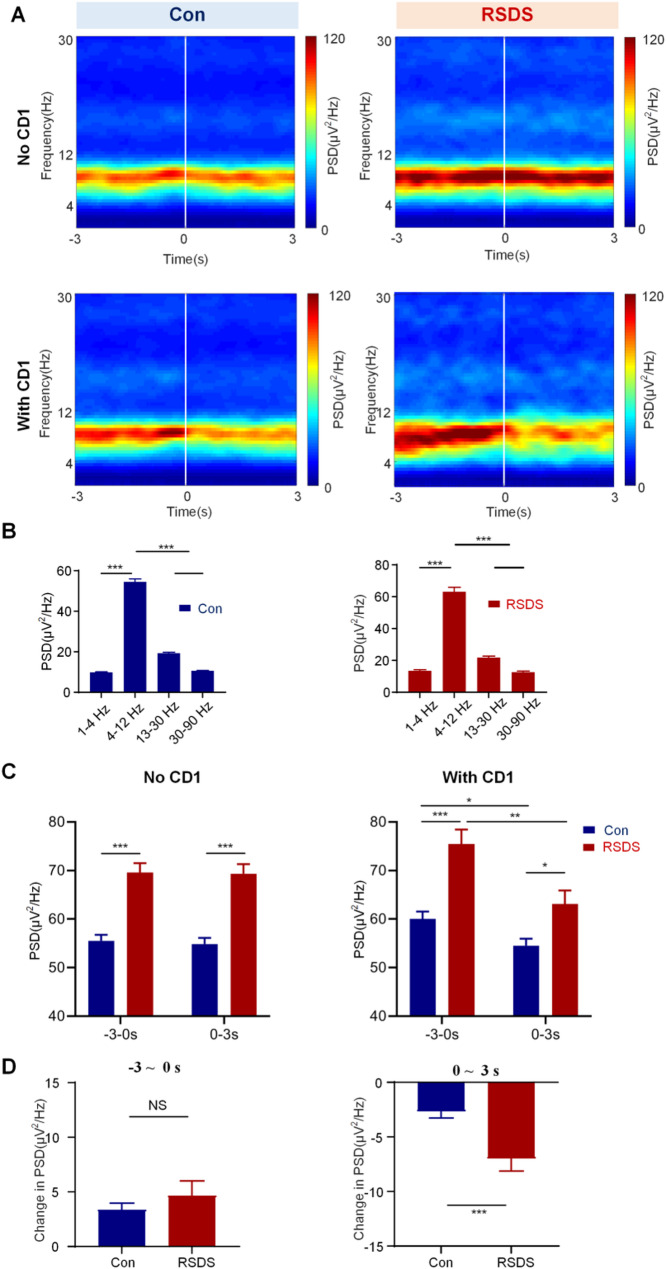
Social defeat alters the theta oscillations in BLA. **A** Time-frequency analysis of BLA during social interaction in control and RSDS groups, with a focus on the transition to the interaction band (−3 to + 3 s). The white line represents the reference point, with 0 marking the moment the mouse enters the interaction zone. The color bars represent strength of PSD. **B** Comparison of PSD in BLA among different frequencies in control and RSDS groups. **C** The PSD of BLA in different stages. Left: No CD1; Right: With CD1. **D** Change in PSD of BLA at different stages. All data are expressed as mean ± SEM. NS, no significant; * *P <* 0.05, **P < 0.01, ***P < 0.001 (Con, *n* = 8; RSDS, *n* = 8)

**Fig. 3:**
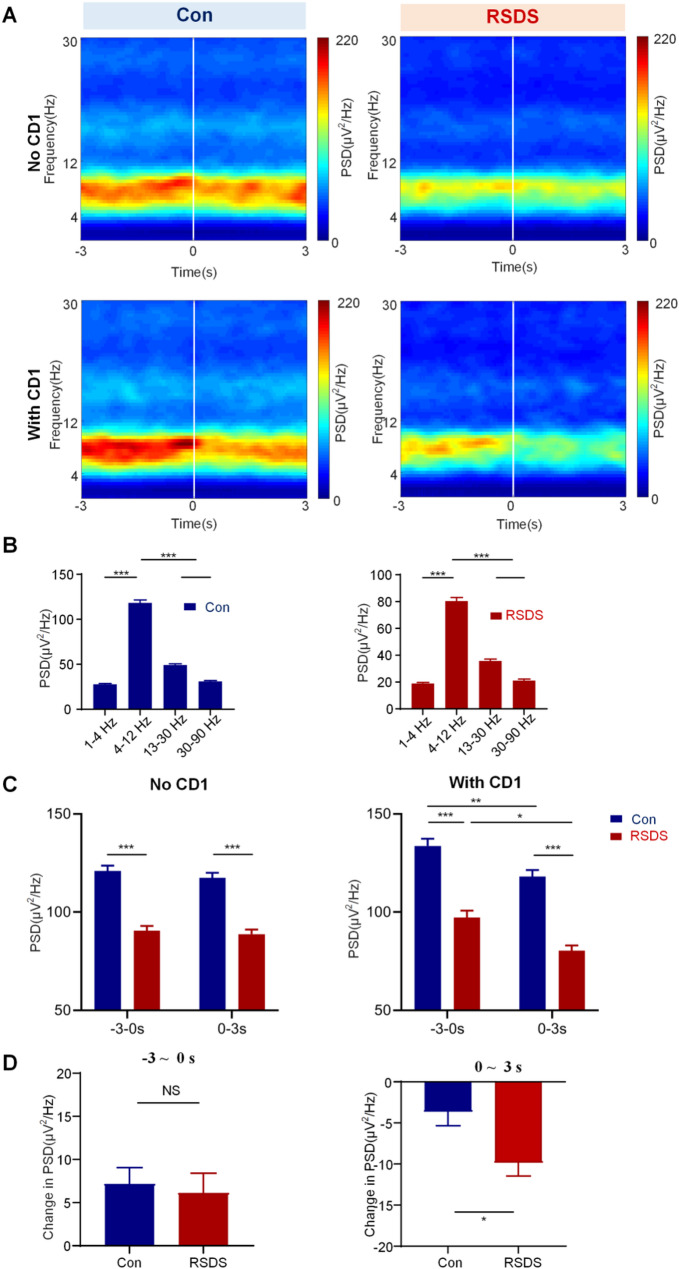
Social defeat alters the theta oscillations in vHPC. **A** Time-frequency analysis of vHPC during social interaction in control and RSDS groups, with a focus on the transition to the interaction band (−3 to + 3 s). The white line represents the reference point, with 0 marking the moment the mouse enters the interaction zone. The color bars represent strength of PSD. **B** Comparison of PSD in vHPC among different frequencies in control and RSDS groups. **C** The PSD of vHPC in different stages. Left: No CD1 phase; Right: With CD1 phase. **D** Change in PSD of vHPC at different stages. All data are expressed as mean ± SEM. NS, no significant; *P < 0.05, **P < 0.01, ***P < 0.001 (Con, *n* = 8; RSDS, *n* = 8)

During social interaction tests, LFP signals in BLA and vHPC were simultaneously recorded using a multi-channel microelectrode array (Fig. 1F and G). Firstly, the LFP signals collected from BLA and vHPC were analyzed by time-frequency analysis. This analysis revealed the PSD was predominantly concentrated in the theta band (4–12 Hz) in both the BLA and vHPC (Figs. 2A and 3A). We performed statistical analysis of the PSD among five frequency bands during With CD1 phase, comparing the control and RSDS groups. The results showed that the PSD in the theta band was significantly higher in both the control and RSDS groups compared to other frequency bands (Figs. 2B and 3B, *P* < 0.001).

Next, to investigate the characteristic phase of LFPs neural oscillations in the BLA and vHPC, we performed a statistical analysis of the PSD at different stages (−3–0s and 0–3 s) during both the No CD1 and With CD1 phases, comparing the control and RSDS groups. During the No CD1 phase, we observed that theta power in the RSDS group was elevated relative to the Control group across both the baseline (–3–0 s stage) and 0–3 s stage (both, *P* < 0.001), suggesting a general altered of baseline theta activity in RSDS mice. Notably, RSDS mice showed reduced theta power in the vHPC but increased theta power in the BLA during the No CD1 phase, demonstrating opposing, region-specific alterations in theta oscillations following social defeat. While within-group comparisons between the two time windows (–3–0 s vs. 0–3 s) showed no significant change in either group (*P* > 0.05). During the With CD1 phase, theta power in the RSDS group was significantly differed than in the control group at both time windows (BLA: − 3–0 s: *P* < 0.001; 0–3 s: *P* < 0.05; vHPC: − 3–0 s: *P* < 0.001; 0–3 s: *P* < 0.001). Moreover, both groups showed a significant change in theta power from baseline (−3–0 s) to (0–3 s) time window (BLA: Con: *P* < 0.05; RSDS: *P* < 0.01; vHPC: Con: *P* < 0.01; RSDS: *P* < 0.05;), indicating dynamic modulation of theta activity during the social interaction. These findings support the view that the With CD1 phase is the characteristic phase in the social interaction test that reveals RSDS-induced alterations in neural oscillations.

Additionally, in the analysis of PSD changes (Change in PSD = PSD_with_CD1_ – PSD_No_CD1_) in the temporal distribution between the − 3–0s and 0–3 s stages during social interaction, we found that no significant difference between the control and the RSDS groups at −3–0s stage (Figs. 2D and 3D, top, BLA: Con 3.367 ± 0.5954, RSDS 4.659 ± 1.352, *P* = 0.3115; vHPC: Con 7.177 ± 1.877, RSDS 6.130 ± 2.271, *P* = 0.7544). However, at the 0–3 s stage, the change in PSD in the RSDS group were significantly higher than those in the control group (Figs. 2D and 3D, down, BLA: Con: −2.659 ± 0.6085, RSDS: −6.981 ± 1.151, *P* < 0.001;vHPC; Con − 3.648 ± 1.700, RSDS − 9.842 ± 1.624, *P* < 0.05). These results suggest that 0–3 s is the characteristic stage of neural oscillation in social defeat in the BLA and vHPC. Overall, These results demonstrate that exposure to social defeat significantly alters the theta oscillatory activity in both the BLA and vHPC.

Moreover, o assess whether theta alterations are specifically related to behavioral vulnerability, we included a group of resilient mice (*n* = 4, SI ratio ≥ 1) in the analysis. Results are shown in the revised Supplementary Materials (Figure S1). We examined theta PSD in the BLA and vHPC during the 0–3 s stage of the With CD1 phase. In the BLA, no significant differences in theta PSD were found between resilient, control, and RSDS-susceptible (SI < 1) groups. Although the resilient group showed a lower mean theta PSD than the susceptible group, this was not statistically significant—likely due to the small sample size. In the vHPC, the resilient group differed significantly from the RSDS-susceptible group, but not from controls. These findings suggest that theta alterations are specifically associated with behavioral vulnerability, rather than general stress exposure.

### SVM decoded social defeat

**Fig. 4:**
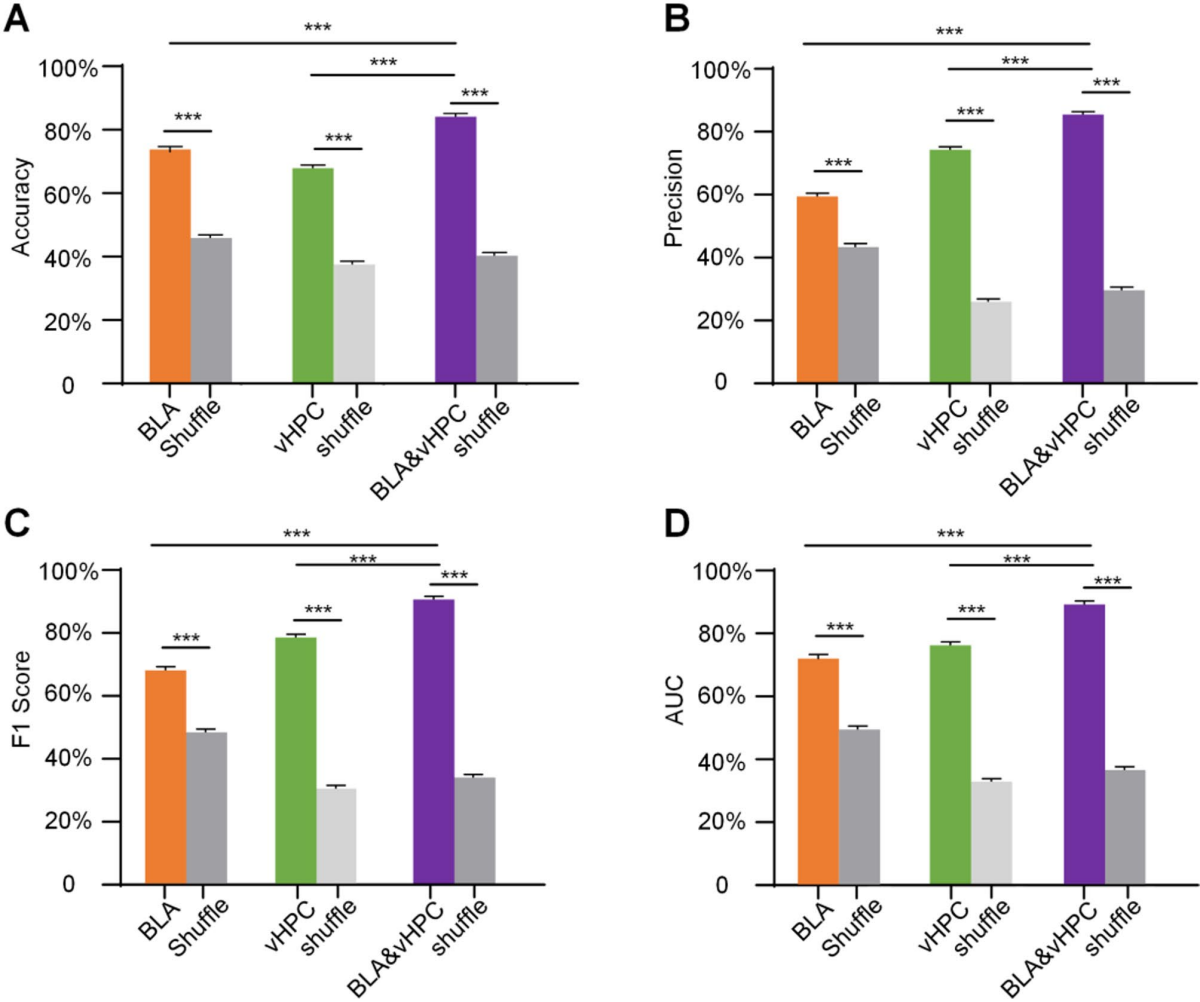
Decoder performance for real data and shuffled data. Decoder accuracy (**A**), precision (**B**), F1 score (**C**) and AUC (**D**) of the real data and the shuffle data. Data are expressed as mean ± SEM. ***P < 0.001

To accurately classify the control and the RSDS groups, we trained a decoder model based on support vector machines. The results demonstrated significant differences in accuracy when comparing real data from the BLA or vHPC alone, as well as from the fusion of both brain regions, to shuffled data. Specifically, the results were as follows: accuracy (BLA: real data = 74.54% ± 0.20%, shuffled data = 46.64% ± 0.27%; vHPC: real data = 68.60% ± 0.25%, shuffled data = 38.28% ± 0.24%; BLA&vHPC: real data = 84.89% ± 0.20%, shuffled data = 41.03% ± 0.26%,, *P* < 0.001; Fig. 4A), precision (BLA: real data = 60.20% ± 0.27%, shuffled data = 44.12% ± 0.30%; vHPC: real data = 75.02% ± 0.23%, shuffled data = 26.69% ± 0.18%; BLA&vHPC: real data = 86.19% ± 0.21%, shuffled data = 30.39% ± 0.23%, *P* < 0.001; Fig. 4B), F1 score (BLA: real data = 62.70% ± 0.33%. shuffled data = 44.70% ± 0.27%; vHPC: real data = 72.38% ± 0.20%, shuffled data = 28.40% ± 0.16%; BLA&vHPC: real data = 83.61% ± 0.21%, shuffled data = 31.85% ± 0.21%, *P* < 0.001; Fig. 4C), and AUC (BLA: real data = 65.77% ± 0.42%, shuffled data = 45.35% ± 0.23%; vHPC: real data = 69.75% ± 0.18%, shuffled data = 30.53% ± 0.12%; BLA&vHPC: real data = 81.22% ± 0.21%, shuffled data = 33.60% ± 0.18%, *P* < 0.001; Fig. 4D). Moreover, the classification performance using fused data from both brain regions was significantly superior to using data from either the BLA or vHPC alone (*P* < 0.001).

### Social defeat alters the theta ATE between BLA and vHPC

**Fig. 5:**
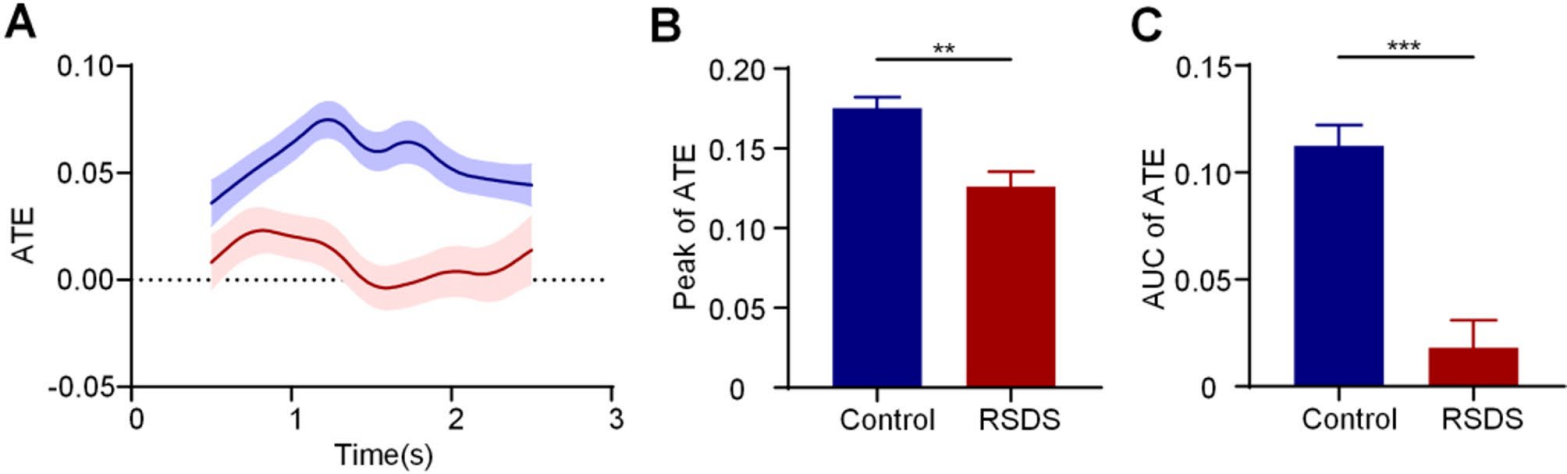
Social defeat alters the theta ATE between BLA and vHPC. **A** ATE distribution between BLA and vHP during the 0–3 s stage of the With CD1 phase. **B** Peak of theta ATE. **C** AUC of theta ATE. Data are expressed as mean ± SEM. ** *P* < 0.01, ***P < 0.001

Given the critical role of theta oscillations in long-range synchronization within the BLA–vHPC circuit during social interaction, we examined changes in amplitude-based information transfer. Specifically, we calculated ATE in the theta frequency band from the BLA to the vHPC during the 0–3 s stage of the With CD1 phase to assess inter-regional communication, as shown in Fig. 5A. We further compared the peak values (Fig. 5B, *P* < 0.01) and the area under the curve (AUC) of the ATE (Fig. 5C, *P* < 0.001) between groups and found that both metrics were significantly lower in the RSDS group compared to the control group.

### Ketamine improved the theta oscillation of BLA and vHPC and alleviated social defeat behavior

**Fig. 6:**
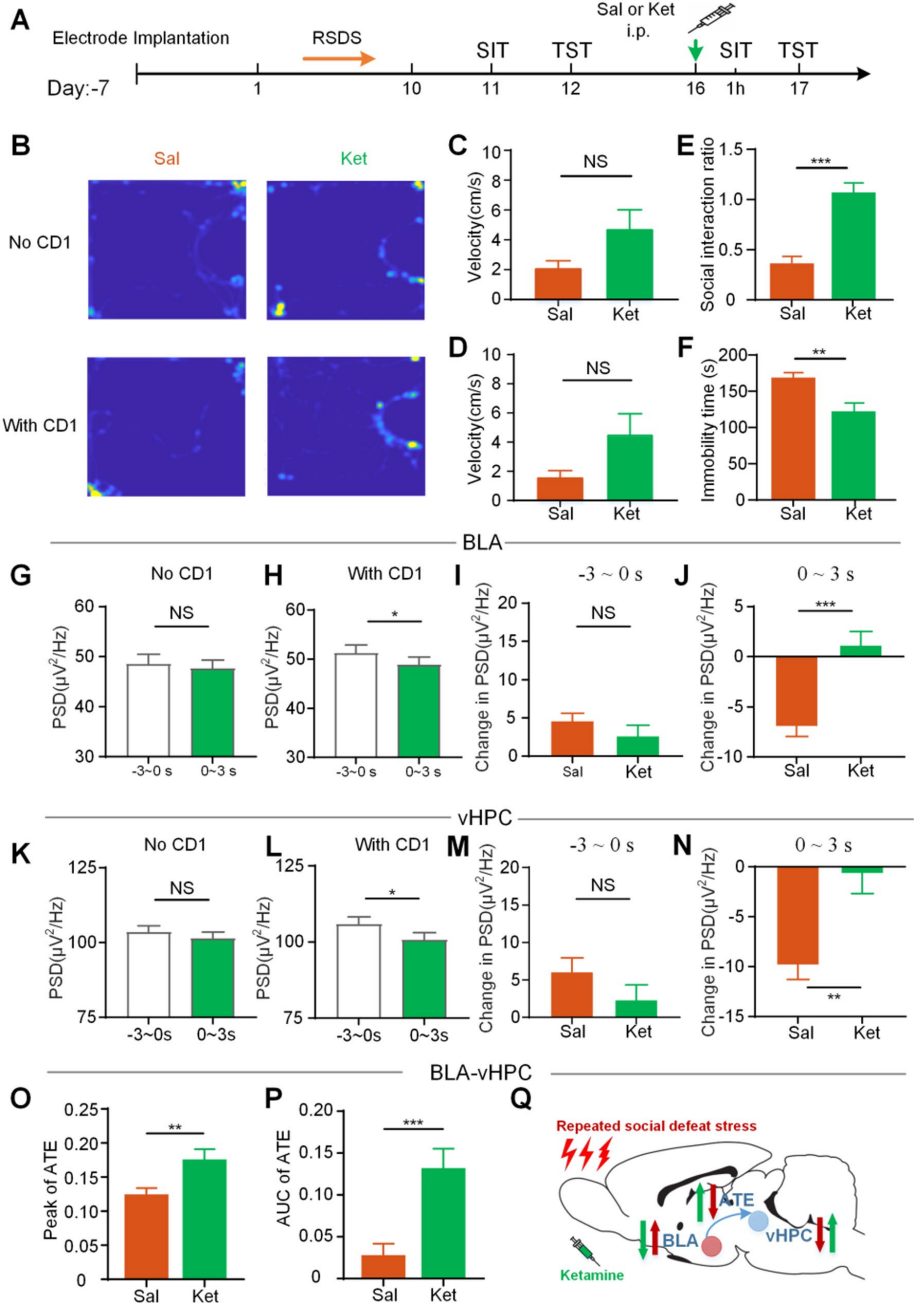
Ketamine alleviates depression-like behaviors and alteres the theta oscillation of BLA and vHPC. **A** Experimental schedule for ketamine administration. **B** Heatmap of the representative trajectory in saline- and ketamine-injected mice during the social interaction tests, with or without CD1 targets. **C, D** Running speed of animals during the social interaction test in the No CD1 phase (C) and the With CD1 phase (D). **E** The social interaction ratio in the interaction zone. **F** The immobility time in TST. **G, H** The theta PSD of BLA at different stages in No CD1 (G) and With CD1 (H). **K, L** The theta PSD of vHPC at different stages in No CD1 (K) and With CD1 (L). **I**,** M** The changes in PSD of BLA (I) and vHPC (M) during the − 3–0s stage in With CD1 phase. **J**,** N** The changes in PSD of BLA (J) and vHPC (N) during the 0–3 s stage in With CD1 phase. **O** Peak of theta ATE. **P** AUC of theta ATE. **Q** Putative model of social defeat altering theta oscillations and behavior, based on experimental and computational evidence. Data are expressed as mean ± SEM. NS, no significant; *P < 0.05, **P < 0.01, ***P < 0.001 (Sal, *n* = 8; Ket, *n* = 8)

Ketamine has been shown to have a powerful, rapid and sustained antidepressant effect [30–32], with its antidepressant activity lasting for at least 24 h [33–35]. To assess whether the LFP oscillatory activities of BLA and vHPC are key factors for social behavior, we administered ketamine to mice. First, we conducted behavioral studies, the social and depression-like behaviors of the mice were measured 1 h and 24 h after ketamine injection (Fig. 6A). One hour after ketamine injection, compared with the saline group, the time spent in the interaction zone during the With CD1 phase of the social interaction test (SIT) was significantly increased (Fig. 6B), and the social interaction ratio also increased markedly (*P* < 0.001; Fig. 6E). However, no significant differences in velocity were observed between the two groups during the No CD1 and With CD1 phases (No CD1: *P* = 0.1029; With CD1: *P* = 0.0883; Fig. 6C and D).

Twenty-four hours after ketamine injection, the immobility time in TST was significantly reduced (*P* < 0.01; Fig. 6F). These results demonstrate that ketamine has the ability to regulate both social and depression-like behaviors in mice. Then, we analyzed the theta oscillation characteristics in the BLA and vHPC. The results showed that there was no significant difference in PSD of BLA and vHPC during the No CD1 phase at −3–0s and 0–3 s stages (Fig. 6G and K, BLA: *P* = 0.4717; vHPC: *P* = 0.39). However, in the With CD1 phase, the PSD at the − 3–0s stage was significantly higher than that at the 0–3 s stage(Fig. 6K and L, *P* < 0.05). During the − 3–0s stage, there was no significant difference in PSD changes between the ketamine and the saline groups in the With CD1 phase (Fig. 6I and J, BLA: *P* = 0.2602; vHPC: *P* = 0.2766), while during the 0–3 s stage, compared with the saline group, the PSD changes of the ketamine group were significantly reduced (BLA: *P* < 0.05; vHPC: *P* < 0.01; Fig. 6M and N). Moreover, ketamine treatment effectively reversed the RSDS-induced reduction in directional information transfer from the BLA to the vHPC, as evidenced by increased ATE values (Fig. 6O–P; peak of ATE: *P* < 0.01, AUC of ATE: *P* < 0.001). A putative model summarizing how social defeat alters theta oscillations and social behavior, based on both experimental and computational findings, is illustrated in Fig. 6Q. These results suggest that ketamine alters the theta oscillation patterns in both the BLA and vHPC in social defeat mice.

## Discussion

In this study, we used in vivo implantation of a multi-channel microelectrode array to record the electrophysiological signals of BLA and vHPC brain regions in social defeat mice during a social interaction test. Following signal acquisition, we performed the neural oscillation analysis using the short-time Fourier transform. This analysis characterized the PSD of BLA and vHPC in RSDS is significantly differed than control in the theta band at the 0–3 s stage in the social interaction zone of the With CD1 phase. Additionally, ketamine was administered to regulate the oscillatory activity in social defeat mice, and the underlying neural mechanisms were further investigated. Our fundings indicate that social defeat resulted in altered theta oscillations in both the BLA and vHPC. RSDS mice exhibited significantly reduced information transfer from BLA to vHPC, while ketamine treatment effectively restored this connectivity. Moreover, ketamine rapidly and effectively reversed these neural oscillatory changes, along with the associated social avoidance behaviors.

The behavioral results indicated that, in the presence of aggressive mice, social defeat mice exhibited a significant decrease in the time spent in the interaction zone, thereby demonstrating substantial social avoidance and depressive-like behavior. This suggests that social defeat mice have developed social behavior disorders, a finding consistent with previous studies [4]. The analysis of power spectral density distribution in different frequency bands revealed that RSDS exerts different effects on BLA and vHPC. Specifically, RSDS resulted in an augmentation of the PSD of BLA. From the perspective of neural mechanisms, this was due to the fact that, in the resting state, BLA was strictly controlled by strong inhibitory constraints, and RSDS eliminated these inhibitory constraints that caused excessive excitation of BLA neurons [16, 36]. Conversely, RSDS led to a decrease in the PSD of vHPC. This is attributable to RSDS inhibiting the BLA-vHPC circuit, which consequently diminished the activity of vHPC neurons, consistent with previous findings [37].

Repeated social defeat stress has been shown to lead to increased BLA activity, with BLA neurons exhibiting heightened sensitivity to afferent nerves following stress [38, 39]. And it also leads to a decrease in the volume of vHPC, resulting in decreased activity [40]. In this study, we have trained a support vector machine-based decoder to classify predictions for the control (naive) and RSDS groups. The results demonstrated that the decoder utilizing the fusion of BLA and vHPC in the PSD performed optimally in terms of classification prediction of the control and RSDS groups. This finding may be attributed to the synergistic role played by BLA and vHPC in RSDS [21, 41]. Hultman et al. employed machine learning techniques to identify a spatiotemporal dynamic neural network capable of predicting major depression-related behavioral impairments in mice subjected to RSDS [42]. The activation pattern within this network propagates through the amygdala and ultimately converges in the ventral hippocampus. The findings of this study align with those of previous related research.Social behavior can be influenced in a bidirectional, direct, but reversible manner between BLA and vHPC [43–45].

In addition, ketamine is a non-competitive N-methyl-D-aspartate receptor (NMDAR) antagonist that can block the ion permeability channels within the NMDAR, thereby exerting a rapid antidepressant effect [46]. Ketamine selectively inhibits the activity of GABAergic interneurons in the marginal region by blocking NMDAR and activating α-amino-3-hydroxy-5-methyl-4-isoxazolepropionic acid (AMPA) receptors. This leads to increased presynaptic glutamate release and modulates theta band oscillatory power, contributing to its antidepressant properties [47–49]. Previous studies have indicated that ketamine primarily exerts its effects through compensatory mechanisms involving global brain network characteristics which ameliorate neural oscillation disruptions induced by RSDS, further supporting its rapid antidepressant action [50].

The present study demonstrates that oscillatory activity in the BLA and vHPC is associated with behavior subsequent to social defeat, and that ketamine has a rapid antidepressant effect on the negative effects of social defeat. These findings offer new insights into the electrophysiological mechanisms underlying behavioral responses to social defeat, highlighting potential therapeutic avenues for addressing depression-related social dysfunction.

## Conclusions

The findings of this study indicate that repeated social defeat stress induces social avoidance and depression-like behaviors. Through the analysis of the power spectrum within the BLA and vHPC, it was observed that the PSD in the theta band (4–12 Hz) in the RSDS group was significantly altered compared to the control group during the 0–3 stage of the With CD1 phase, when mice entered the social interaction zone. Furthermore, machine learning-based analysis successfully distinguished between the control and RSDS groups based on neural oscillatory activity in the BLA and vHPC. Treatment with ketamine alleviated social avoidance and depression-like behaviors in RSDS mice and enhanced theta oscillations in both the BLA and vHPC. These results suggest that social defeat stress modulates theta oscillations in the BLA and vHPC, highlighting potential therapeutic avenues for addressing depression-related social dysfunction.

## Supplementary Information

Supplementary Material 1.

## Data Availability

The datasets used and analysed during the current study are available from the corresponding author on reasonable request.
