# Supplementary material for: Altered theta oscillations in basolateral amygdala and ventral hippocampus related to social defeat
Source: BMC Neurosci. 2025 Aug 27;26:53. doi: 10.1186/s12868-025-00972-6 (PMC12392519; doi:10.1186/s12868-025-00972-6)
Supplement: Supplementary file 1 — Supplementary Material 1. [file 12868_2025_972_MOESM1_ESM.docx]

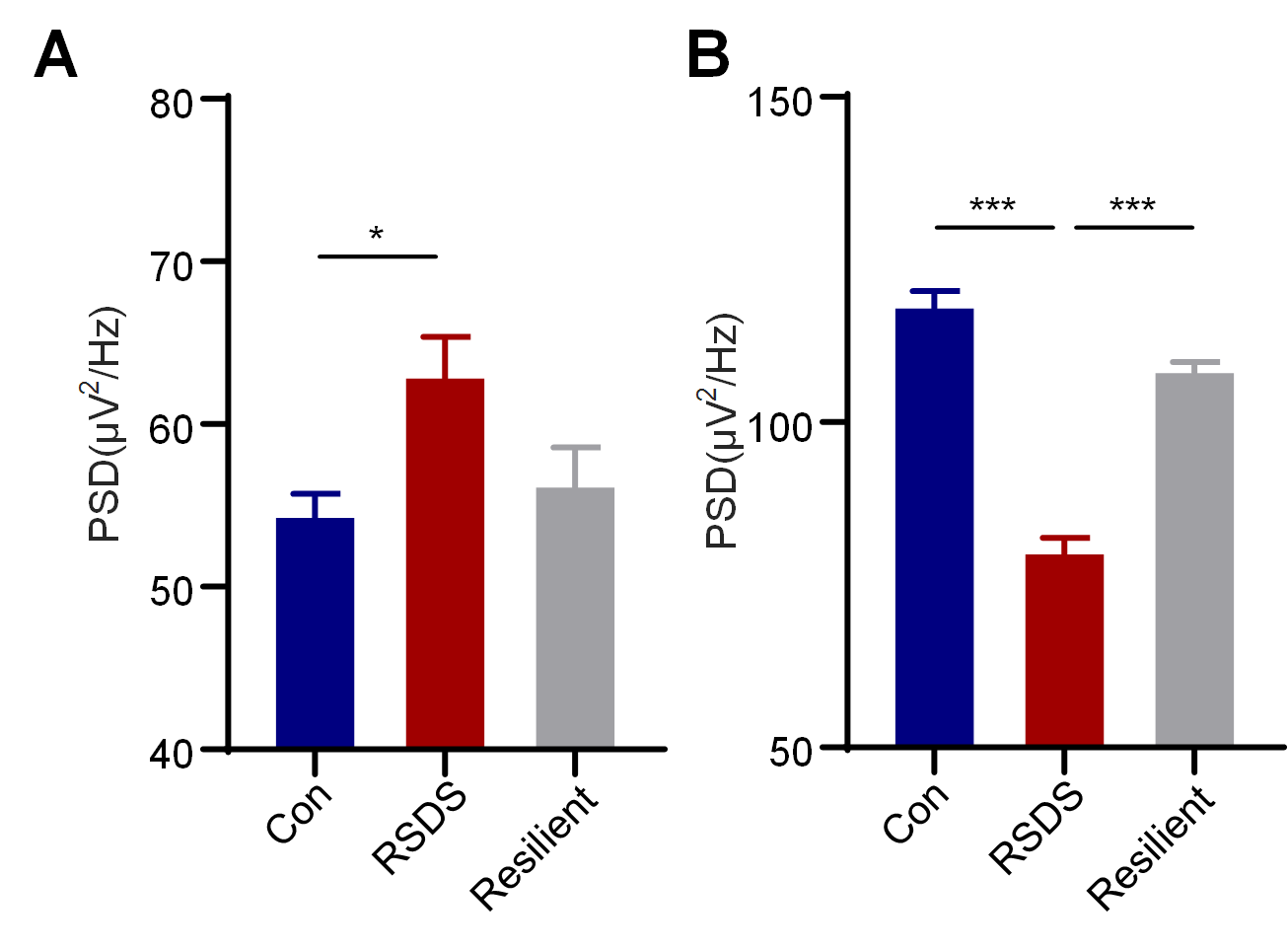


**Figure S1. Social defeat selectively alters the theta oscillations in BLA and vHPC during the With CD1 phase (0–3 s stage). (A)** PSD comparison of theta oscillations in the BLA across Control, RSDS-susceptible, and Resilient groups. **(B)** PSD comparison in the vHPC. All data are expressed as mean ± SEM. * *P* < 0.05, ****P* < 0.001 (Con, n=8; RSDS, n= 8; Resilient n = 4).
